# Single-Molecule Time-Resolved Spectroscopy in a Tunable STM Nanocavity

**DOI:** 10.1021/acs.nanolett.3c04314

**Published:** 2024-01-29

**Authors:** Jiří Doležal, Amandeep Sagwal, Rodrigo Cezar de Campos Ferreira, Martin Švec

**Affiliations:** † Institute of Physics, Czech Academy of Sciences; Cukrovarnická 10/112, CZ16200 Praha 6, Czech Republic; ‡ Faculty of Mathematics and Physics, Charles University; Ke Karlovu 3, CZ12116 Praha 2, Czech Republic; § Institute of Organic Chemistry and Biochemistry, Czech Academy of Sciences, Flemingovo náměstí 542/2, CZ16000 Praha 6, Czech Republic

**Keywords:** TEPL, STM, photoluminescence, nanocavity, TCSPC, phthalocyanine

## Abstract

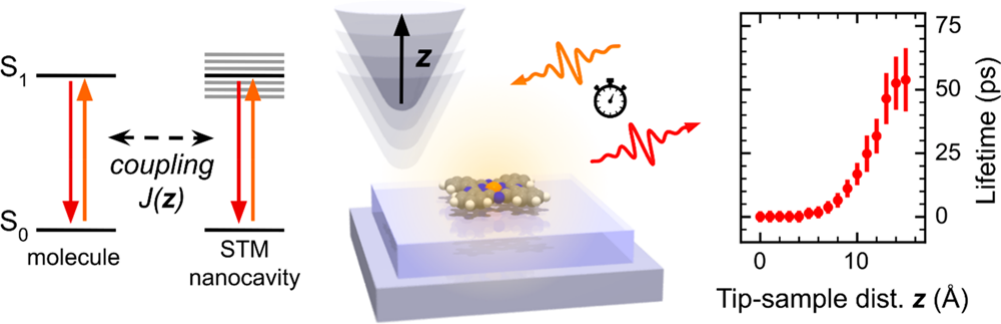

Spontaneous fluorescence rates of single-molecule emitters are typically on the order of nanoseconds. However, coupling them with plasmonic nanostructures can substantially increase their fluorescence yields. The confinement between a tip and sample in a scanning tunneling microscope creates a tunable nanocavity, an ideal platform for exploring the yields and excitation decay rates of single-molecule emitters, depending on their coupling strength to the nanocavity. With such a setup, we determine the excitation lifetimes from the direct time-resolved measurements of phthalocyanine fluorescence decays, decoupled from the metal substrates by ultrathin NaCl layers. We find that when the tip is approached to single molecules, their lifetimes are reduced to the picosecond range due to the effect of coupling with the tip-sample nanocavity. On the other hand, ensembles of the adsorbed molecules measured without the nanocavity manifest nanosecond-range lifetimes. This approach overcomes the drawbacks associated with the estimation of lifetimes for single molecules from their respective emission line widths.

Coupling single-molecule emitters with the electromagnetic field of an optical cavity can stimulate their excitation and radiative decay rates, increasing the fluorescence yields and bringing them closer to applications in the GHz range. Such enhancement is often realized in the vicinity of plasmonic structures of subwavelength size such as sharp metal tips,^1^ colloids,^2^ or bowtie nanoantennas,^3^ where the electric field is strongly localized and intensified. In the regime of weak coupling between the emitter and the cavity, the Purcell effect^4^ will cause shortening of the exciton lifetime, broadening, and a red-shift of the emission line (known as the Lamb shift).^5^ On the other hand, in the limit of strong coupling, energy is coherently exchanged between the molecule and the plasmon. This has also been demonstrated in nanoparticle-on-mirror^6^ experiments by detecting characteristic Rabi oscillations and the emission line splitting.^7^ However, it remains a challenge to achieve control over the coupling, exact geometry of the molecule in the nanocavity, and the resulting behavior. The scanning tunneling microscope (STM) provides an ideal platform, where positioning of the molecules within the nanocavity formed by the tip and a substrate can be performed with outstanding precision. STM combined with an optical detection scheme represents a prospective methodology that can provide benchmarks of the effect of coupling on the emission of single chromophores and fundamental insights into the area of the light–matter interaction.

The first experimental attempts to measure the exciton lifetime from the electroluminescence of single molecules in STM nanocavity utilized a second-order photon correlation technique, the Hanbury–Brown–Twiss interferometry, which confirmed the expected single-photon character of the emission and found an exponential decay half-life on the order of hundreds of picoseconds.^8,9^ Similar results have been obtained with the RF-phase fluorometry.^10^ However, in these experimental setups, it was not possible to distinguish the exciton decay rates from the dynamics of charge carrier transport preceding the formation of the excitons.^11,12^ In other reports, excitation lifetime values were estimated from the emission line widths, setting them in the range of hundreds of femtoseconds.^13,14^ Also, this approach has limitations, stemming from possible exciton–phonon coupling occurring particularly in chirally adsorbed molecules,^15^ breaking of Kasha’s rule,^16,17^ hot luminescence, and instrument resolution limits, which generally lead to a secondary apparent peak broadening, obscuring the intrinsic effect of the lifetime.

Therefore, a direct method to excite an emitter and measure its decay rate as a function of the nanocavity geometry would be more adequate, in order to exclude the charge carrier dynamics and spectral line broadening effects and to distinguish the role of the far-field contributions to the signal.^18^ We use the STM with optical excitation and detection capabilities to perform direct time-correlated single photon counting (TCSPC) using photoluminescence (PL) of single zinc– and magnesium–phthalocyanines (ZnPc, MgPc). With this technique, we could determine the excitation lifetimes of the molecule in the tunable nanocavity and make a comparison between the far-field photoluminescence, focused to achieve micrometer spatial resolution (μPL) and near-field tip-enhanced photoluminescence (TEPL) with single-molecule resolution, showing a dramatic effect of the electromagnetic field confinement on the lifetime.

For the PL experiments, we prepared systems with ultralow concentrations of MgPc and ZnPc species adsorbed on 2–3 ML NaCl/Ag(111) and on clean Ag(111). The morphology of the as-prepared NaCl/Ag(111) substrate consists of three different major landscapes, i.e., the 3 ML and 2 ML NaCl islands and the bare Ag(111).^11,14,19−22^ In the μPL (far-field) experiments performed by irradiating these samples directly with a focused laser beam of 1.96 eV energy photons (schematically depicted in Figure 1a), we observe fluorescence signals only from the systems with NaCl decoupling layers, whereas the molecules on bare Ag(111) do not contribute (Figure S1) because of nonradiative quenching of the excitations to the substrate.^23^ The MgPc signature in μPL appears as a single emission line at 1.90 eV with 4 meV full width at half-maximum (see Figure 1c) and can be attributed to the transition from the excited state to the ground state. In the case of ZnPc, a peak with a similar line width is observed at 1.91 eV, and another much narrower peak at a lower energy of 1.88 eV appears. The former peak broadening can be attributed to the exciton–libron coupling as described in our previous study.^22^ In contrast, the peak at 1.88 eV is very narrow and might originate from molecules in symmetrical configurations, e.g., adsorbed at Na^+^ sites or pinned to defects. Because the estimated number of molecules on NaCl illuminated by the focused laser light is between 10^4^ and 10^5^, one would also expect the influence of inhomogeneous broadening^24^ stemming from nonequivalent adsorption configuration of each molecule in the ensemble due to incommensurability of NaCl overlayer with the Ag(111) and local variations in the electronic properties of the substrate. Our energy resolution puts its energy range below 0.3 meV, in agreement with previous observations.^24,25^

**Figure 1:**
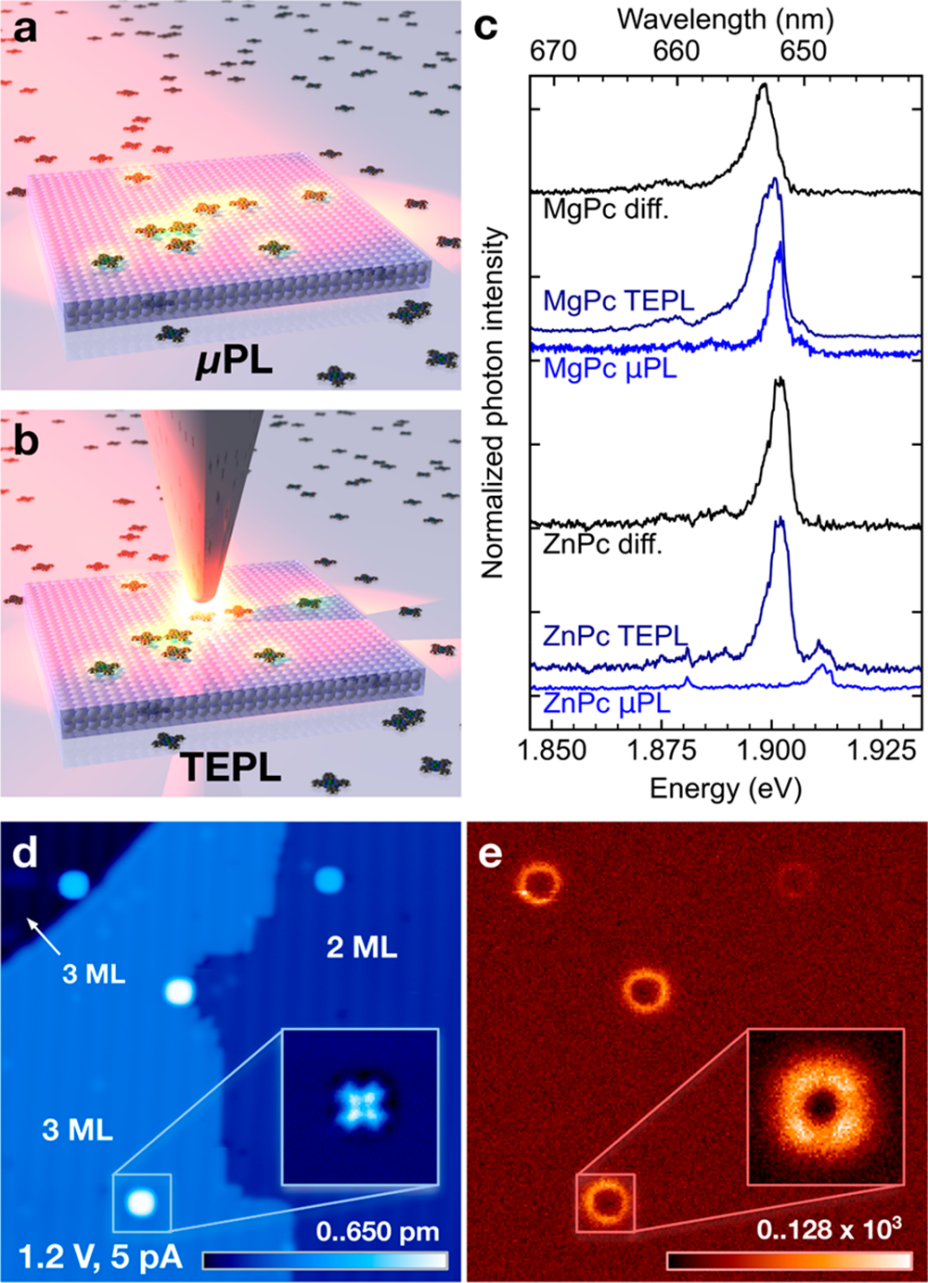
Schemes of (a) the far-field μPL experiment with molecules on a decoupling layer and (b) the near-field + far-field TEPL experiment of a single molecule. (c) Comparison of the μPL, TEPL, and difference spectra for the Mg- and ZnPc adsorbed on 2–3 ML NaCl/Ag(111). The MgPc TEPL spectrum was measured at 1.2 V and 1.7 pA set point, and the μPL spectrum was obtained with the tip retracted 1 nm from the set point. The ZnPc TEPL was measured at 1.2 V and 5 pA set point, and the μPL spectrum was obtained with the tip retracted 120 nm from the set point. (d) STM topography of MgPc/2–3 ML NaCl/Ag(111), size 44 × 44 nm^2^, tunneling conditions 1.2 V, 5 pA, 3 ms per pixel. (e) APD map of the identical area, taken simultaneously and rebinned from 512 × 512 to 256 × 256 pixels for better contrast. The insets in (d, e) are taken simultaneously in the constant-height mode in the transport gap at −0.7 V, maximum current 1 pA, and total laser power 100 μW, resulting in peak rate 130 × 10^3^ events/point, image size 4 × 4 nm^2^, and the original APD map rebinned from 128 × 128 to 64 × 64 pixels.

When the distance between the atomically sharp STM tip apex and a molecule is closed well below a few nanometers, new plasmonic modes are created in the nanocavity gap between the Ag tip and the surface (see Figures S1 and S2). They mediate an efficient bidirectional coupling between the electromagnetic near-field of the nanocavity coupled to the molecular emitter and the far-field of the irradiation and detection beams, thus permitting excitation and detection of single-molecule PL for the species that are electronically detached from the metal substrate (Figure 1b). These conditions define the TEPL mode, which is however also inherently including the μPL contributions due to the diffraction-limited size of the focused laser spot. With the tip in the vicinity of the MgPc or ZnPc molecules adsorbed on 3 ML NaCl, characteristic PL peaks emerge near the 1.9 eV mark. These relatively broad peaks are apparently superimposed over their corresponding μPL backgrounds that can be subtracted in order to get the single-molecule contributions originating in their respective S_1_–S_0_ transitions, as described previously.^26^ The individual molecule emission peaks are red-shifted for both Mg- and ZnPc (by 3 and 10 meV, respectively) and also broadened compared to the μPL measurements, in agreement with previous reports.^11^ The shapes of the spectra do not bear any hallmarks of a strong coupling regime in accord with the previous evaluation.^27^

The spatial distribution of the TEPL yield can be visualized because the μPL background signal is independent of the lateral nanocavity position over the sample. We recorded the photon rate by an APD filtered to a spectral range of 642–662 nm while scanning the tip over the NaCl-covered sample area, using the STM topography mode. The STM image presented in Figure 1d shows a step between 3 and 2 ML of NaCl/Ag(111) with a few MgPc molecules distributed in the scanned area. The corresponding APD photon map in Figure 1e shows the PL of the molecules as hollow circular features, corresponding to a single molecule each. These features are well-pronounced at 3 ML NaCl, but they are at the limit of detection at 2 ML. This is due to a significantly higher degree of the electronic decoupling of the molecules from the substrate at 3 ML.^9,28,29^ A detailed constant-height mapping with the APD (Figure 1e, inset) reveals a characteristic donut-shaped pattern of the emission with hints of four lobes. This appearance has been observed in the previous work and explained theoretically by variation of coupling between the emitter electronic transition density and the nanocavity.^11,30^ We have not observed any TEPL signal from the molecules adsorbed directly on Ag(111) (see Figure S1) due to the nonradiative quenching of their excited states by the substrate.^23^ It has been reported that at very close tip–sample distances, in particular when the molecule is contacted or even lifted up with the tip, the Raman signal^31−34^ can be dramatically enhanced on NaCl or bare metal substrates. However, these conditions were deliberately avoided in our study.

We employ APD and a pulsed laser to perform TCSPC of PL on the phthalocyanines in both TEPL and μPL regimes, as shown in the scheme in Figure 2a. High pulse repetition rates of the laser allow us to obtain good statistics of the photon-arrival delays, recorded and binned to histograms using a fast pulse counter connected to the APD, and synchronized with the laser seed pulse. The spectral region of the APD measurement was filtered to the 649–673 nm region of the Mg/ZnPc main emission lines. The dependence of the MgPc decay rates on the vertical tip–sample displacement (Δ*z*) in Figure 2b reveals a dramatic change in the character of the time traces. At large tip–sample distances in the μPL regime, they have a single-step shape indicative of a surprisingly long exponential decay, whereas with the tip in the vicinity of the molecule in the TEPL regime, a very fast decay appears on top at the step onset. Such a rise in intensity is a clear indication that the PL enhancement effect takes place due to the intensification of the electromagnetic field within the narrowing nanocavity gap.

**Figure 2:**
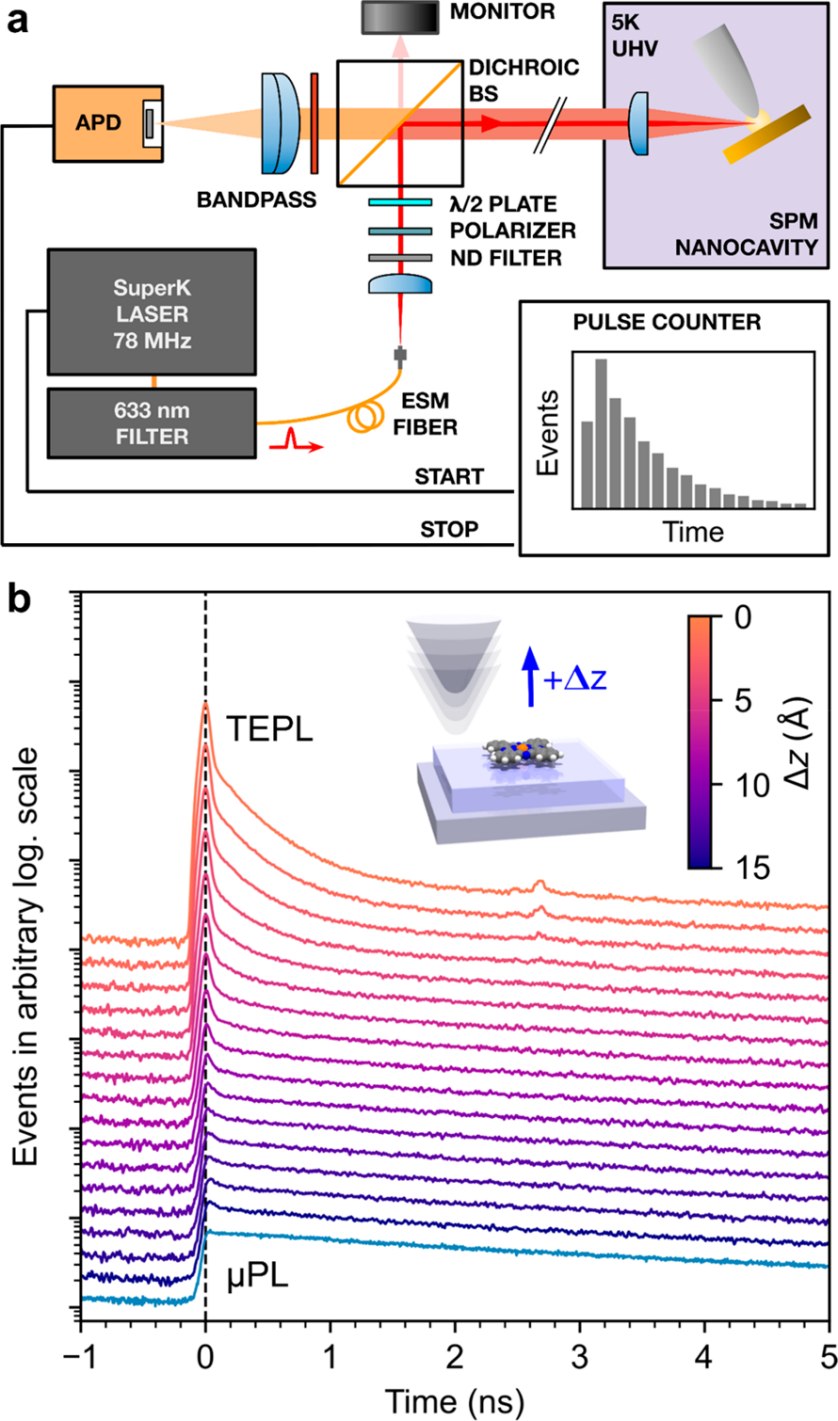
(a) Scheme of the TCSPC setup for the measurement of the transition between μPL and TEPL regimes with a tunable nanocavity using a pulsed laser and an avalanche photon detector (APD). (b) MgPc S_1_–S_0_ PL emission photon-arrival time histograms as a function of the decreasing tip–sample distance (order top to bottom), crossing from the μPL to the TEPL regime, measured at lateral distance of 1.5 nm from the center of the molecule (to avoid mixing with Raman signal). The Δ*z* = 0 set point was set at tunneling conditions of 1.2 V and 80 pA on NaCl. The bottom curve in the graph is the μPL background without the TEPL contribution, measured with the tip away from the molecule. The total laser power for the measurements was 100 μW, and the averaging time was 180 s per spectrum. The feature emerging at around 2.8 ns at the closest tip–sample distance originates from a weak spurious reflection in the optical path.

The time traces measured in the μPL regime for both considered chromophores manifest a long fluorescence decay spanning a few nanoseconds (Figure 3). The portions of the traces before the onset of the μPL show spurious peaks originating from the internal reflections of the optical setup, independent of the PL signal. They are relatively strong in the case of ZnPc which has an inherently weaker PL response. As the reflection decays are very fast and placed well before the onset of the molecular PL, they can be excluded from the analysis of the decay rates. Fitting of the acquired time histograms was done with a sum of two exponential functions with different decay rates, convolved with our instrument-response function (IRF), and achieved a good match. The IRF was obtained on the clean Ag(111) areas close to the measured molecules by measuring the decay of the photoinduced nanocavity plasmon (Figure 4a), which is considered to be significantly faster than the molecular emission; the typical electronic dephasing lifetimes are <100 fs.^10^ The need for fitting two decay constants (τ_1_ and τ_2_) suggests that two different processes contribute to the total fluorescence yield.

**Figure 3:**
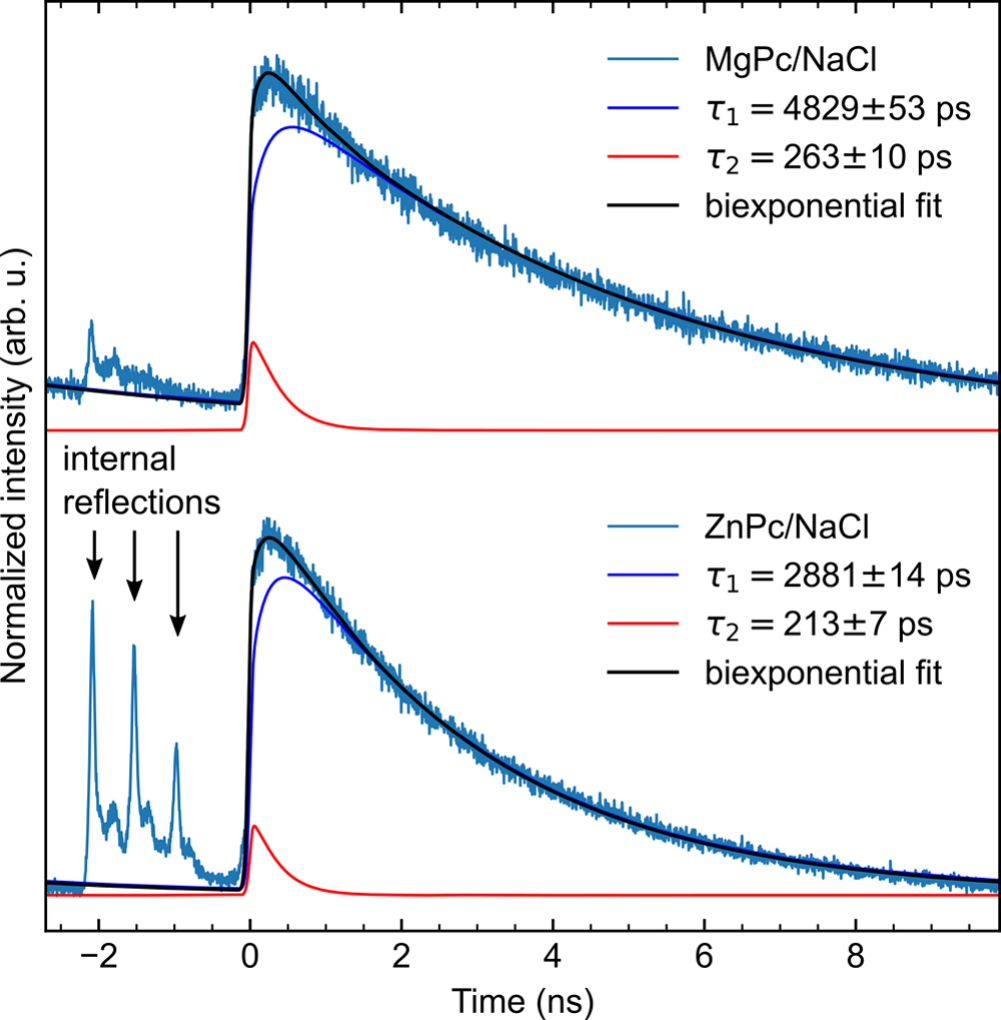
Biexponential fit of the μPL photon-arrival histograms with a retracted tip for (top) the S_1_–S_0_ emission of MgPc, total laser power 280 μW, averaging time 180 s, and (bottom) for the S_1_–S_0_ emission of ZnPc, total laser power 600 μW, averaging time 900 s. The zero time is set to the onsets of the μPL from the system. Spurious peaks before the onsets of the μPL (denoted by black arrows) are attributed to the internal scattering and reflections of the exciting light in the optical setup.

**Figure 4:**
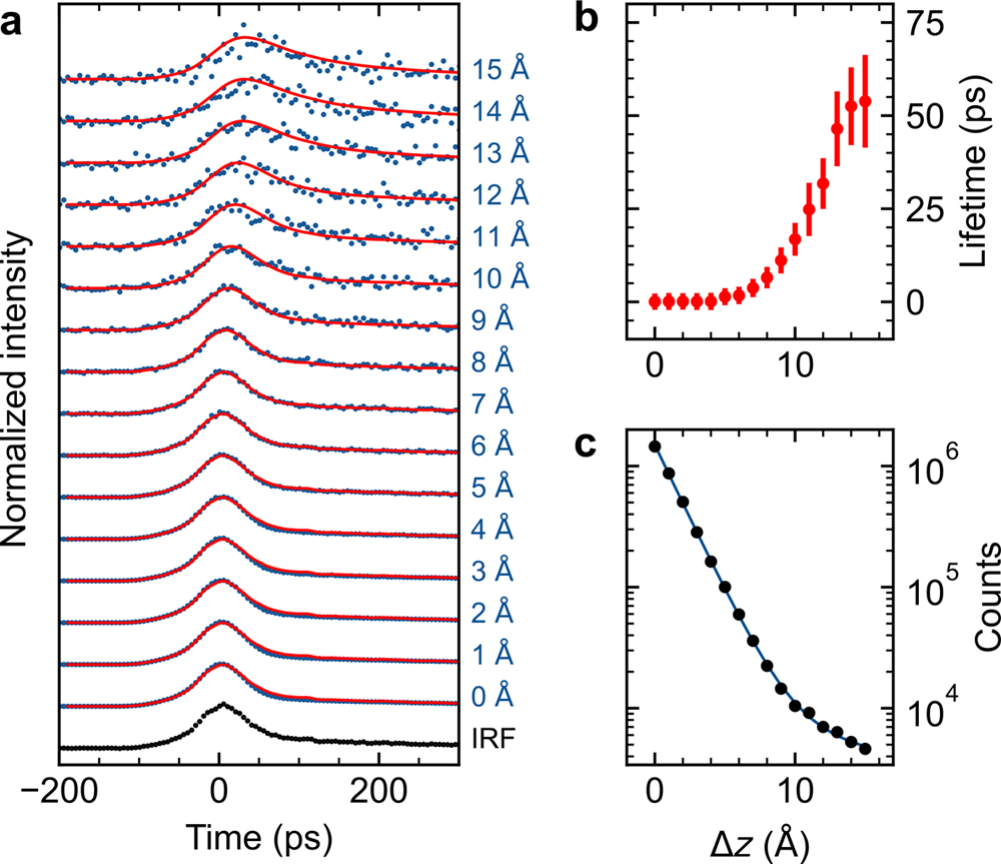
(a) Details of the Δ*z* dependence of the TCSPC measured on the S_1_–S_0_ emission of a single MgPc from Figure 2b after subtraction of the μPL background (measured away from the molecule) and intensity normalization. The IRF (black) is used for the iterative reconvolution of the fluorescence data with a monoexponential decay. (b) Exciton lifetime obtained from the monoexponential fit as a function of relative tip–molecule distance. (c) Sum TEPL intensity obtained from the signal in (a) as a function of Δ*z* (black dots) fitted with a biexponential function (blue curve).

The partial yield corresponding to the longer-lived process, responsible for the majority of the signal, we attribute to the molecules adsorbed on top of the 3 ML NaCl, because of their prevalence in the studied system and consequently better decoupling from the metal substrate. The lifetimes of 3 ns for ZnPc and 5 ns for MgPc obtained from the fits are close to the values reported for molecules in solutions^35^ and are nearly an order of magnitude longer than self-decoupled tetrapodal perylene on Au(111), measured in a similar manner.^36^ On the other hand, the contribution with a shorter exciton lifetime is possibly associated with molecules on 2 ML NaCl, where a stronger nonradiative quenching is expected to limit the lifetime.^23^ The shorter-lifetime contribution to the total emission is significantly weaker in comparison with the longer-lifetime yield. The assignment of the dominant components of the signal to the molecules on 2–3 ML NaCl is supported by a control experiment performed on molecular aggregates (see Supporting Note 3). The generally long lifetimes indicate that even relatively small separation from the metal is sufficient to suppress the nonradiative losses (extended discussion in Supporting Note 4).

For obtaining the excitation lifetimes of single molecules located in the nanocavity from the TEPL measurements, the fitting has to be performed on the fast-decay signal without the contribution of the μPL background. Therefore, we have first subtracted the μPL background, measured at Δ*z* = 15 Å at a clean NaCl region, from all the decay curves in the data set and fitted the resulting data with a convolution of an exponential decay and the IRF, as shown in Figure 4a. At higher Δ*z* there is a clear departure of the single-molecule PL peak position and a visibly longer temporal spread compared to the IRF (a simulated behavior is presented in Figure S4). This is reflected in the fitted values of the exponential decay rates in Figure 4b, which can be directly interpreted as the exciton lifetimes of the MgPc. The exciton lifetimes are staying well below the precision limit of the fit (error bars show standard deviation, estimated by the procedure described in Supporting Note 6) for Δ*z* distances up to 6 Å, and rapidly rise for higher Δ*z*, reaching values in excess of 50 ps at 15 Å, where we approach our detection limit of the TEPL. The values obtained for the lifetimes at closer tip–molecule distances are consistent with the value determined by resonant-absorption STM-PL of the H_2_Pc S_1_–S_0_ transition,^17^ which gives a peak width between 0.2 and 0.9 meV corresponding to a 0.7–3.3 ps lifetime, and point out a strong enhancement effect of the nanocavity. In addition, we observe an exponential Δ*z* dependence of the TEPL sum yield (evaluated as total counts for each trace) from the single MgPc. An abrupt increase of the exponential decay factor (approximately 13×) occurs at Δ*z* = 10 Å, indicating a transition from a picocavity regime attributed to a single atom at the tip apex and the more delocalized nanocavity given by the radius of curvature of the tip as described by Yang et al.^11^

In conclusion, by using direct TCSPC measurements on single molecular emitters placed on decoupling layers and comparing it to the measurements performed in μPL regime, we have verified the strong increase of radiative decay rate in a tunable plasmonic nanocavity gap in STM and the associated lifetime shortening, yet without any signature of the strong coupling regime. We observed and quantified the exciton lifetime in the STM nanocavity for the MgPc species on 3 ML NaCl as a function of the tip–sample distance. Also, in the μPL regime, we have found that the intrinsic lifetimes of the emitter ensembles on the surface correspond to their lifetimes, typically found in solutions. Therefore, a pathway is paved toward combined mesoscopic and nanoscopic measurements of excitons of molecular chromophores, their aggregates, and other quantum emitters. We envisage future experiments to determine the lifetimes with greater precision using pump-and-probe schemes or GHz modulation of the incident light.

## Methods

### Sample Preparation and Optical Setup

The NaCl was evaporated at 610 °C on half of a clean Ag(111) surface kept at 110 °C by thermal deposition. This leads to the sample being partially covered with NaCl islands typically 400–10000 nm^2^ large of various thicknesses (in the typical proportion of 70% 2 ML, 28% 3 ML, very low amounts of 4 ML, and traces of ≥5 ML). ZnPc or MgPc molecules were evaporated at 382 °C on the sample held at 10 K, achieving a concentration of around 40 molecules per 100 × 100 nm^2^. The excitation source in the PL and the TCSPC measurements was a pulsed supercontinuum laser (Fianium SuperK) with a spectral filtering module (Varia SuperK). Its center wavelength was set to 633 nm, with a 10 nm bandwidth. The laser output beam, from an endlessly multimode fiber, was collimated by a lens and filtered again using a 633/25 nm bandpass filter, sent through a rotatable half-wave plate, and polarized to the plane intersecting the axis of the SPM tip. The beam was reflected into the UHV system by a Semrock 633 nm RazorEdge dichroic long-pass beamsplitter and focused by an internal lens of the Createc 4K SPM instrument into the tunneling junction area. The final focused laser spot had an approximate diameter of 10 μm, which illuminates an ellipse with 10 μm minor and 20 μm major axes at a 60° angle of incidence (to the surface normal) and thus defines the spatial resolution of the μPL regime. The PL spectra were measured by an Andor Kymera 328i spectrograph with a 1200 grooves/mm, 500 nm blaze grating.

For the TCSPC measurements, photon arrival time detection was performed using one MPD PDM Series-100 single-photon avalanche detector with a specified 35 ps jitter together with the Swabian Instruments Time Tagger Ultra pulse counter. The bin size was set to 4 ps (ZnPc data) or 5 ps (MgPc data). The photon-arrival histograms were accumulated for 180, 360, and 900 s in the measurements of ZnPc near-field, nanocavity plasmons, and ZnPc far-field, respectively.

### Fitting Procedure

For all the μPL and TEPL TCSPC measurements, a corresponding IRF was determined by measuring a plasmonic response of the Ag(111) surface with the tip in the tunneling regime and subtracting the μPL background with the tip retracted by 1.5 nm. To eliminate the spectral dependence of the detector’s temporal response, the same optical filter as for μPL and TEPL was used. Fitting of the μPL data was done with the biexponential function  convolved with the IRF. For the fitting of the TEPL dependence on Δ*z*, a convolution of monoexponential decay with IRF was used. The μPL fits were performed on the entire range of the time window, excluding the internal reflections region before the onset of the PL response of the molecules. The TEPL fits were performed in the range between −100 and 305 ps, sufficient for lifetimes <100 ps (see Figure S4). The dependence of the total counts on the Δ*z* biexponential function was used in the form  using logarithmic weighting factors. The Python package LMfit was used for the data fitting.

### TEPL Tuning

For TEPL measurements, we used tips made of a 25 μm diameter Ag wire sharpened by head-on sputtering with a focused ion beam. Further cleaning by Ar^+^ sputtering was done before insertion into the microscope. The nanocavity plasmon resonance was tuned by voltage pulses and controlled tip indentations into the Ag(111). An effective coupling of the incident laser beam to the nanocavity is typically evidenced by a broad feature in the emission spectrum^34^ (see Figures S1 and S2) or as a rigid shift of the field emission resonances, corresponding to the energy of the excitation source.^37^

## Supporting Information Available

Additional figures showing μPL of MgPc/Ag(111); tip-induced electroluminescence and TEPL gap plasmons an Ag(111); μPL and STM of an annealed MgPc/NaCl/Ag(111) sample; discussion on the μPL lifetime and origin; simulated convolution of a Gaussian IRF with the exponential decay; error analysis; supporting references (PDF)
