# Supplementary material for: Single-Molecule Time-Resolved Spectroscopy in a Tunable STM Nanocavity
Source: Nano Lett. 2024 Jan 29;24(5):1629–34. doi: 10.1021/acs.nanolett.3c04314 (PMC10853955; doi:10.1021/acs.nanolett.3c04314)
Supplement: Supplementary file 1 — nl3c04314_si_001.pdf [file nl3c04314_si_001.pdf]

Supporting Information for
Single-molecule time-resolved spectroscopy in a tunable STM nanocavity

Jiří Doležal[1#*], Amandeep Sagwal[1,2], Rodrigo Cezar de Campos Ferreira[1], Martin Švec[1,3*]

[1] Institute of Physics, Czech Academy of Sciences; Cukrovarnická 10/112, CZ16200 Praha 6, Czech Republic

[2] Faculty of Mathematics and Physics, Charles University; Ke Karlovu 3, CZ12116 Praha 2, Czech Republic

[3] Institute of Organic Chemistry and Biochemistry, Czech Academy of Sciences; Flemingovo náměstí 542/2. CZ16000 Praha 6, Czech Republic

[#] Present address: Institute of Physics, École Polytechnique Fédérale de Lausanne, CH-1015 Lausanne, Switzerland

*Corresponding authors: dolezalj@fzu.cz, svec@fzu.cz

Supporting Note 1: μPL measurements of the MgPc/Ag(111)

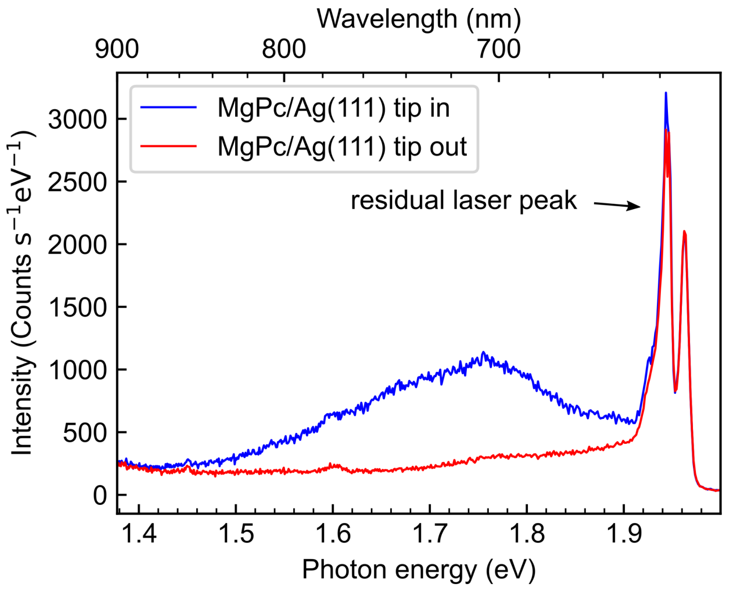

Figure S1: μPL measurement of Ag(111) substrates with <0.1 ML MgPc coverage. The measurements have been performed with 150 gr./mm grating and 10 nm laser bandwidth. No molecular luminescence peak has been observed. The difference between the curves is a photo-induced gap plasmon.

Supporting Note 2: Electroluminescence characterization of the plasmonic spectra of the tips

For the experiments, the tips have been modified in order to achieve a good bidirectional coupling of the excitation and photoluminescence of the molecules. Their plasmonic response has been measured by electroluminescence and is presented in Figure S2.

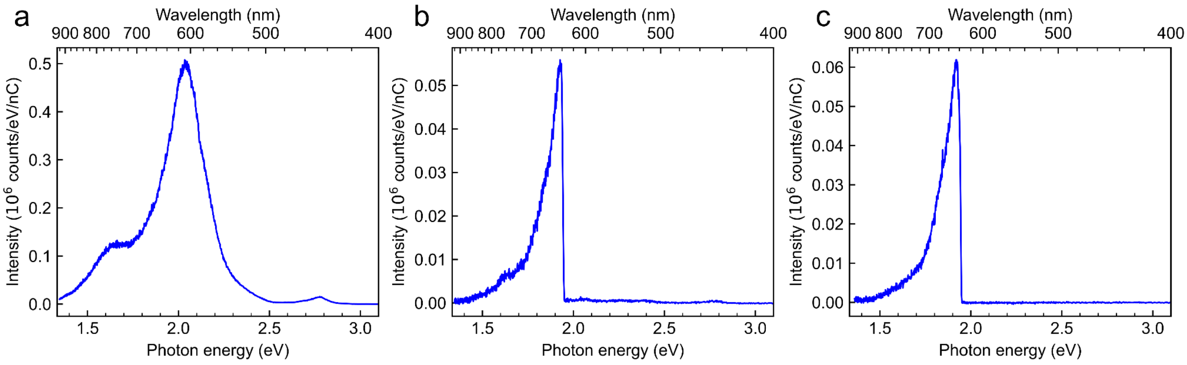

Figure S2: a) full EL spectrum of the tip used for ZnPc TEPL. b) EL spectrum of the same tip, measured after passing through the edge filter and attenuated by the 90:10 beamsplitter. c) EL spectrum of the tip on Ag(111), used for MgPc TEPL, measured after passing through the edge filter and attenuated by the 90:10 beamsplitter. All spectra have been measured at 2.5 V, 10 s and 1 nA on Ag(111).

Supporting Note 3: µPL and TCSPC at annealed MgPc/NaCl/Ag(111)

We perform a control experiment to exclude the possibility that the slow decay histograms in the µPL mode originate from any well-decoupled molecules on thicker NaCl layers (≥ 4 ML), that sporadically exist at the studied systems and might contribute strongly enough. To this end, we have used one of the samples with the MgPc molecules (after finishing all the single-molecule measurements) and allowed the temperature to reach 100 K for 2 min, in order to form molecular aggregates. These can be formed only on sufficiently extended areas encompassing enough chromophore units, as on 2 ML or 3 ML NaCl, but very unlikely on the small rarely appearing patches of the 4 ML or thicker layers, because of their relatively small area and correspondingly low numbers of molecules available for aggregation. The temperature of 100K is not sufficient to allow diffusion of the molecules between areas of different thicknesses of NaCl.

Once the aggregates were formed, several new peaks at different energies appeared in the µPL spectrum (see Figure S3a) and the overall lifetime decreased, which can be understood in terms of molecular self-quenching[1] or stronger average transition dipole moment of the aggregates.[2,3] This supports the interpretation that the majority of the signal on the original unheated samples originates from the emitters at the 2 and 3 ML spacer.

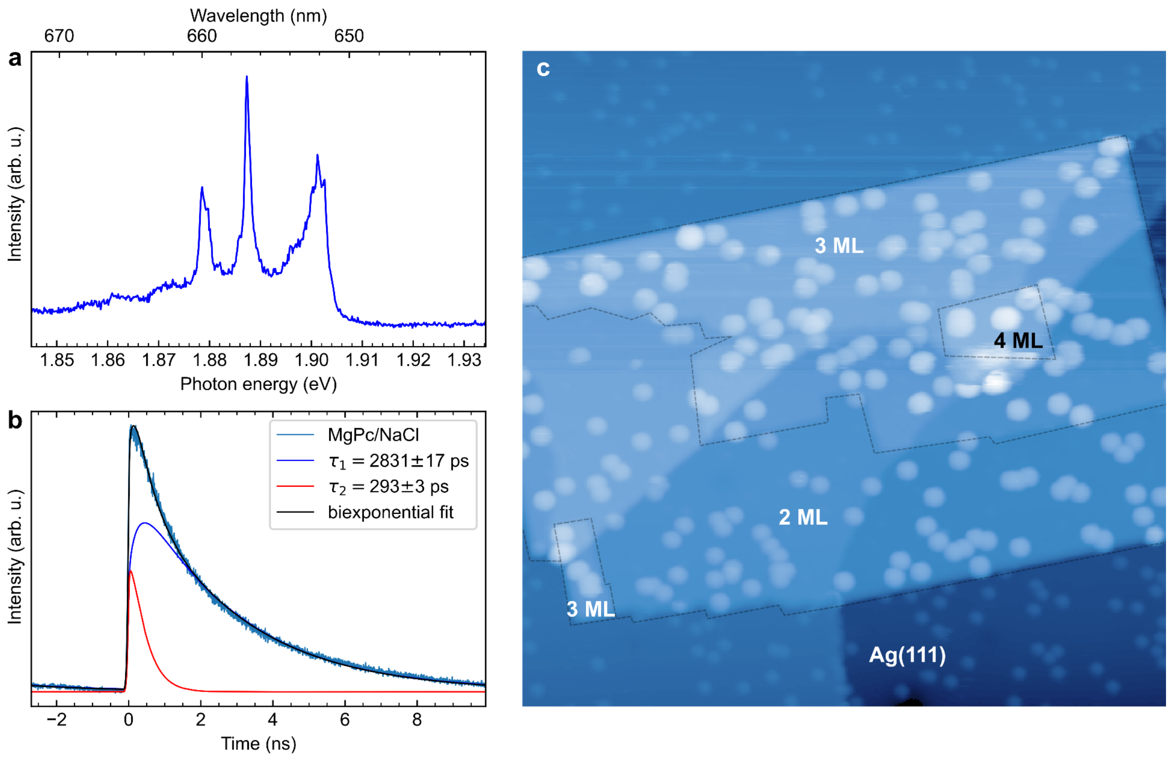

Figure S3: a) µPL of the annealed MgPc/NaCl/Ag(111) sample. A characteristic monomer peak at 1.9 eV, broadened by the libron-exciton coupling, is accompanied by red-shifted peaks originating from randomly formed molecular aggregates. b) Photoluminescence emission photon-arrival time histogram from the same sample shows a general shortening of the lifetimes as demonstrated by the biexponential fit. In this case, the biexponential fitting is only used as an example, as it cannot capture all the possible aggregate configurations on the sample. c) The corresponding STM image of the annealed sample 120 x 120 nm^2, measured at 1.2 V, 5 pA with marked thickness of NaCl.

Supporting Note 4: Discussion of the role of the metal in the vicinity of the molecules

It is quite surprising that the longer exciton lifetimes of the molecules detected by the µPL on thin NaCl insulating layers approach the lifetimes determined in solutions, despite still being in a relative vicinity of the metal substrate. From a previous theoretical study[4], which deals with this problem, it follows that the calculated rate of resonant electron energy transfer between a ZnPP molecule (similar to ZnP on 2-3 ML NaCl and the underlying metal (Cu100) substrate is around 15 µeV for 2 ML and 1.5 µeV for 3 ML, which corresponds to exciton lifetimes of 43 and 430 ps, respectively. Our measurements show an order of magnitude higher values for both ZnPc and MgPc. We cannot fully discard the possibility that the measured decay curves might originate from molecules on 3 and 4 ML, although the control experiments (see Supporting Note 3) indicate against it. The classical electrodynamic calculations of a parallel molecular dipole separated from the metallic sample by an insulating spacer layer, as presented in the theoretical study[4], predict a nonradiative quenching rate (by Ohmic losses) for the thicknesses between 2-5 ML on the order of 3-20 µeV, corresponding to 20-300 ps. At this point, we are inclined to consider the calculated losses as overestimated, since the experimentally measured lifetime of self-decoupled tetrapodal perylene molecules on Au(111) has been also reported considerably higher, i.e. at 600 ps.[5] In this case, the emitter units were separated from the substrate by about 1 nm.

Their standing-up configuration naturally permits a significantly better dipolar coupling to the metal compared to the parallel configuration of ZnPc/NaCl/Ag(111) (see dashed blue vs. dashed gray line in Figure 4a in Ref.[4]). In this sense, the experimental lifetimes in the far-field regime, can be considered as indicative of the rapidly diminishing role of the metal with the decoupling layer thickness.

Supporting Note 5: Simulated convolution of a Gaussian IRF with the exponential decay

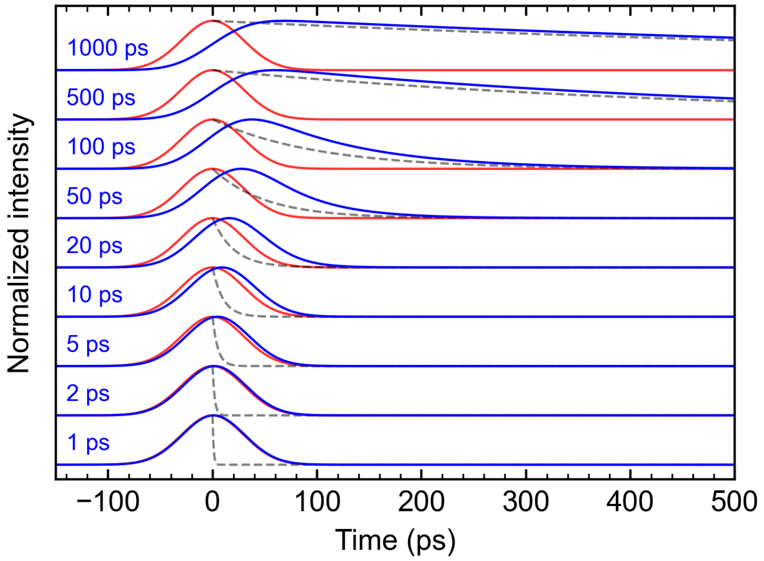

Figure S4: Simulation of the fluorescence decay histograms (blue line) with a given Gaussian IRF (red line) and its full width at half maximum (w_{IRF} = 70 ps), convolved with an exponential function of variable decay rate τ (grey dashed line).

Supporting Note 6: Analysis of the exciton lifetimes fitting errors

The process of deconvolution of the signal with a predetermined IRF (more appropriately called iterative reconvolution) was extensively discussed and experimentally applied[6,7,8] and the conclusion was reached that it is possible to determine fluorescence lifetimes of a single decay comparable to or significantly lower than IRF's FWHM. The limiting factor is the statistical noise which determines the signal/noise ratio (increasing as $1/\sqrt{N}$, where N is the total number of counts). This requires measuring the IRF in the same spectral range as the exciton signal, which is also fulfilled in our case, thanks to the available plasmonic response of the metal substrate and to perform a thorough error analysis of the results.
There are various sources of errors contributing to the total error bars plotted in Figure 4b. The first one corresponds to the variation of the mean arrival time of the signal over the period of the measurement.
In Figure S5a we show a set of data from the measurement session, where the z-dependence shown in Figure 4 was acquired, with various fast decay curves (TEPL data from MgPc for Δz < 4 Å, Tip-enhanced Raman spectroscopy (TERS) data from MgPc and IRF measured as Ag(111) gap plasmon). The mean time (μ) and the full width at half maximum (w) of the IRF in Figure 4 were obtained by fitting a Gaussian function, with an

additive background to account for the nonlinear temporal response of the detector, represented by an exponential decay factor τ_{IRF}: (A_1 exp(-2.773(t-μ)^2/w^2) + A_2 exp(-2.773(t-μ)^2/w^2 $*$ exp(-t/τ_{IRF})). There is no systematic trend in the μ over time and the standard deviation of the mean arrival time of all the data points is σ_{time} = 1.3 ps. This time uncertainty is a result of both distance variation of our optical path (light travels 300 μm in 1 ps in air/vacuum), and the time variation of laser, detector and electronics, including nonlinear response for different signal intensities.

The fitting in Fig 4. relies on subtracting the μPL background from the signal and fitting it by convolution of the IRF and exponential decay (decay factor τ, corresponding to the lifetime), i.e. uses 3 spectra, measured each with their own time uncertainty (σ_{time}). The iterative reconvolution does not allow direct evaluation of the uncertainties of the best-fit parameters if the lifetime τ is below 0.5 ps ($\Delta z < 4$ Å). This is due to the convergence of the fitting parameter τ to its lower set bound (0.01 ps) and the subsequent impossibility of the fitting error estimation. This is likely the hard limit of the employed fitting procedure to detect short lifetimes. In such case, the error of our estimation is given by the standard deviations of the signal arrival time σ_{time_IRF} and σ_{time_signal} for IRF and the total signal, respectively. The standard deviations of the background arrival time can be neglected for the τ < 0.5 ps case, since the exciton signal is very strong compared to the background and the overall noise level is negligible.

The largest source of error, however, comes from the statistical noise and background subtraction for low-intensity signals measured far from the molecule $\Delta z > 4$ Å (see Figure S6) and consequently from the uncertainty in the fitting parameters. To account for this and to provide a realistic estimate of the maximum total errors, we performed a fitting of data with additive noise: i) We randomly shifted the time axis of IRF, signal and background data using normal distribution centered around zero with σ = 1.3 ps. ii) We added a random statistical noise with σ_i = $\max(1, \sqrt{N_i'})$ to the data after background subtraction (we neglect the background noise), where N'_i is the signal value at point i of the histogram after subtraction. In this way, we generated 1000 histograms of the photon-arrival time for each Δz in Figure 4, performed the fitting for each of them and obtained a set of fitted lifetimes τ (see Figure S6, where τ is plotted as a function of background time shift). Finally, we calculated the standard deviation σ_{sim} of the fitted τ for each Δz. The total maximum estimated error plotted in Figure 4b is $\sigma_{tot} = \sqrt{\sigma^2_{sim} + \sigma^2_{fit} + \sigma^2_{time\ IRF} + \sigma^2_{time\ signal}}$ where σ_{fit} is the standard error of fitted parameter τ given by the fitting procedure. Inclusion $\sigma_{time\ IRF}$ and $\sigma_{time\ signal}$ is substantiated by the underestimation of σ_{sim} for the fits where the randomized shifting of the time axis creates limit cases where the fitting result of τ < 0.5 ps approaches the lower bound of the fit. For larger values of τ, the squares of σ_{time_IRF} and σ_{time_signal} are not a major contribution to the σ_{tot}. As explained above, for τ < 0.5 ps the error of the fitting parameter and the simulation could not be obtained. The total error is then defined by $\sigma_{tot} = \sqrt{\sigma^2_{time\ IRF} + \sigma^2_{time\ signal}} = \sqrt{2} \times 1.3$ ps $= 2.4$ ps, which is the lower limit of the error bar plotted in Figure 4 and hence the resolution of our method.

From the shape of the spread of points in Figure S6 one can clearly see that there is a trend for the extracted lifetime values as a function of the background time shift parameter. However, using a conservatively high confidence interval of $\pm 3\sigma$ for the possible total error σ_{tot}, for any $\Delta z > 8$ Å the τ value is well above zero. Therefore we are assured that the

lifetime z-dependence is real.

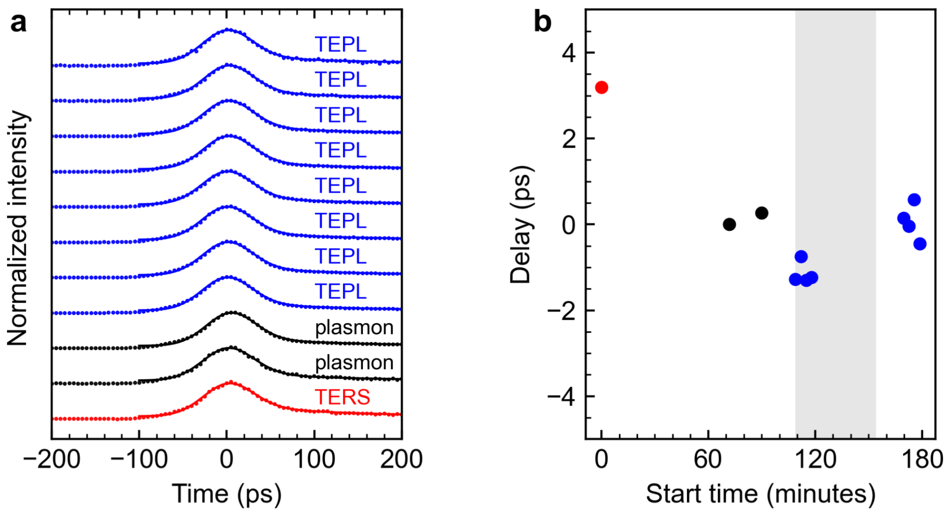

Figure S5. a) Fast-decay TEPL measured at close tip-sample distances (blue), TERS response measured above a molecule (red) and plasmon taken at the substrate (black) TCSPC histograms shown in the chronological order and their corresponding fits with the modified Gaussian. b) Mean time (μ) extracted from the fitting procedure described in ***Supporting Note 6*** *as a function of start time of the measurement. The grey-shaded area represents the time interval where the Δz dependence shown in Figure 4 was measured.*

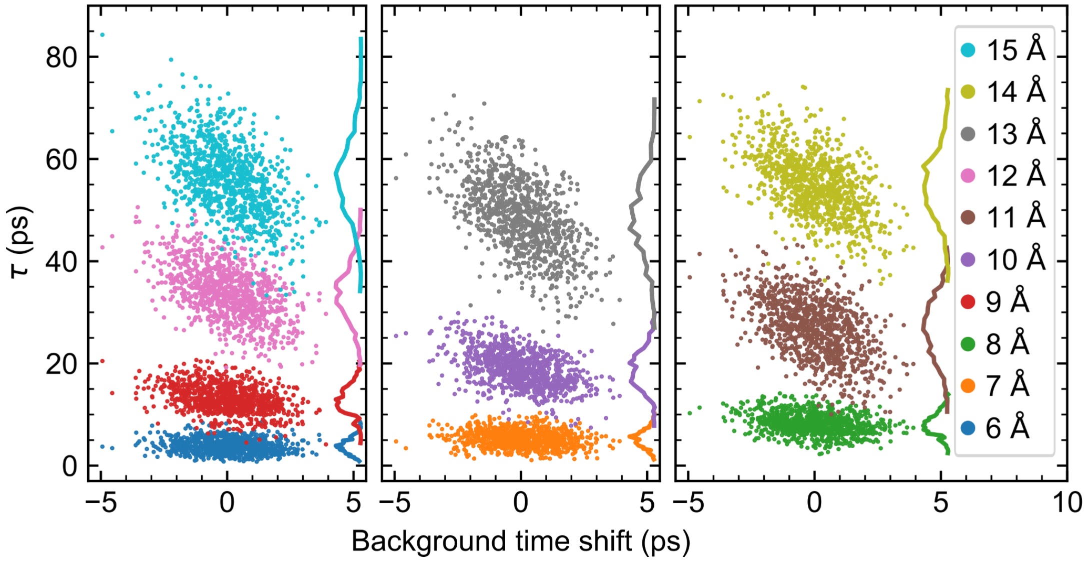

Figure S6: Lifetime values extracted from the fits of the data in Figure 4a with added random artificial noise and random time shift around zero with σ = 1.3 ps to the signal, background signal and IRF plotted as a function of background time shift. The probability distribution of the fitting parameter τ is plotted on the right y-axis with solid lines.

Supporting references

1. Tamošiūnaitė, J., Streckaitė, S., Chmeliov, J., Valkunas, L. & Gelzinis, A. Concentration quenching of fluorescence in thin films of zinc-phthalocyanine. *Chem. Phys.* **572**, 111949 (2023).
2. Zhang, Y. *et al.* Visualizing coherent intermolecular dipole-dipole coupling in real space. *Nature* **531**, 623–627 (2016).
3. Luo, Y. *et al.* Electrically Driven Single-Photon Superradiance from Molecular Chains in a Plasmonic Nanocavity. *Phys. Rev. Lett.* **122**, 233901 (2019).
4. Aguilar-Galindo, F., Zapata-Herrera, M., Díaz-Tendero, S., Aizpurua, J. & Borisov, A. G. Effect of a dielectric spacer on electronic and electromagnetic interactions at play in molecular exciton decay at surfaces and in plasmonic gaps. *ACS Photonics* **8**, 3495–3505 (2021).
5. Ijaz, T. *et al.* Self-decoupled tetrapodal perylene molecules for luminescence studies of isolated emitters on Au(111). *Appl. Phys. Lett.* **115**, 173101 (2019).
6. Smith, D. A., McKenzie, G., Jones, A. C. & Smith, T. A. Analysis of time-correlated single photon counting data: a comparative evaluation of deterministic and probabilistic approaches. *Methods Appl Fluoresc* **5**, 042001 (2017).
7. O’Connor, D. V., Ware, W. R. & Andre, J. C. Deconvolution of fluorescence decay curves. A critical comparison of techniques. *J. Phys. Chem.* **83**, 1333–1343 (1979).
8. O’Connor, D. & Phillips, D. *Time-correlated Single Photon Counting*. (Academic Press, 1984).
